# Discovery and Verification of an Immune-Related Gene Pairs Signature for Predicting Prognosis in Head and Neck Squamous Cell Carcinoma

**DOI:** 10.3389/fgene.2021.654657

**Published:** 2021-05-24

**Authors:** Jiqiang He, Xinqi Fang, Mingming Han

**Affiliations:** ^1^Department of Hand and Microsurgery, Xiangya Hospital of Central South University, Changsha, China; ^2^Department of Neurosurgery, Huashan Hospital, Fudan University, Shanghai, China; ^3^Department of Anesthesiology, The First Affiliated Hospital of USTC, University of Science and Technology of China, Hefei, China

**Keywords:** immune-related gene pairs, head and neck squamous cell carcinomas, the Cancer Genome Atlas, gene expression omnibus, prognosis, tumor immunology

## Abstract

The study of IRGPs to construct the prognostic signature in head and neck squamous cell carcinoma (HNSCC) has not yet elucidated. The objective of this study was to explore a novel model to predict the prognosis of HNSCC patients. The Cancer Genome Atlas (TCGA) and Gene Expression Omnibus (GEO) datasets were set as training and validation cohorts, respectively. The least absolute shrinkage and selection operator (LASSO) and time-dependent ROC were employed to screen the highest frequency immune-related gene pairs (IRGPs) and their best cut-off value. Survival analysis, Cox regression analysis were applied to discover the effects of selected IRGPs signature on survival outcomes. The immune cell proportions were deconvoluted by the CIBERSORT method. After a couple of filtering, we obtained 22 highest frequency IRGPs. The overall survival time of HNSCC patients with a high score of IRGPs was shorter as compared to the ones with a low score in two independent datasets (*P* < 0.001). Six kinds of immune cells were found to be differentially distributed in the two different risk groups of HNSCC patients (*P* < 0.001). GO and GSEA analysis showed these differentially expressed genes enriched in multiple molecular functions. The new IRGPs signature probably confers a new insight into the prognosis prediction of HNSCC patients.

## Introduction

Head and neck squamous cell carcinoma (HNSCC) is one of the most common cancers globally, and more than 600,000 cases are diagnosed annually (Leemans et al., 2011). Smoking, heavy alcohol, and HPV infection are the main potential causes of HNSCC development. Local or distant metastasis is the major factor for cancer-associated death (Huang et al., 2018). Despite multimodal treatments, such as surgery, chemoradiotherapy, and targeted therapies, the 5-year survival rate of HNSCC patients remains poor (Li et al., 2020; Zhang et al., 2020). Interestingly, even patients with the same clinical symptoms, TNM stage, and treatments would have different prognoses (Karam and Raben, 2019). Therefore, the inaccurate effects of the TNM stage on predicting the prognosis of HNSCC patients lead to a clinically meaningful puzzle to explore and develop some novel and better molecular models.

Immunity is a disease defense system composed of a series of biological structures and processes in an organism (Montecino-Rodriguez et al., 2013). It is composed of various proteins, cells, organs, and tissues. Disorders of the immune system can lead to a variety of diseases (O’Byrne and Dalgleish, 2001; de Visser et al., 2006). Many studies have confirmed that the immune system plays an important role in the occurrence and development of tumors. Charlotte Lecerf et al. (2019) recently found OX40L, PDGFRB, and PD-1 act as the potential prognostic biomarkers and major therapeutic targets. Moreover, blockade to the PD1/PD-L1 axis has been approved by the FDA and shows groundbreaking progress in improving HNSCC patients’ survival time (Tsangaris et al., 1969). Discovering and understanding the relationship between immune-related genes and HNSCC could be crucial.

The tumor microenvironment (TME) includes various cell types and extracellular components, such as vasculature, cancer-associated fibroblasts, immune cells, tumor-associated endothelial cells, and extracellular matrix (Wu and Dai, 2017). Accumulated studies show that TME influences therapeutic response and clinical outcomes. Regulation of TME by CTX/L-NIL could improve the sensitivity of tumor cells of chemoradiotherapy resistance (Hanoteau et al., 2019). The expression level of PD-L1 in exosomes of HNSCC correlated with the clinical outcome of patients. The blocking of PD-L1^+^exosomes weakens immune suppression in HNSCC (Theodoraki et al., 2018).

Previous studies showed many gene-expression-based models for predicting cancer patient prognosis (Resteghini et al., 2018). Unfortunately, none of them could be implemented in routine clinical practice because of some issues like overfitting on small discovery data sets and lack of sufficient validation. With more and more large-scale gene expression profiles published online, it could identify potentially more reliable prognosis-related biomarkers in HNSCC. However, these diverse large-scale data also represent a daunting challenge. For instance, the traditional method to normalize gene expression is a difficult task given the potential biological heterogeneity among data sets and technical biases across measurement platforms. Instead, the relative ranking of gene expression may be a good way to eliminate the requirement for data scaling and normalization (Li et al., 2017), producing a robust result in various applications.

Considering that the important roles of immune-related genes, there are emerging studies that calculate the score of immune-related gene pairs (IRGPs) based on its relative raking and report a significant correlation between IRGPs and the prognosis of cancer, including hepatocellular carcinoma, colorectal cancer, gastric cancer, lung adenocarcinoma and cervical cancer (Wu et al., 2019; Zhang et al., 2019; Nie et al., 2020; Sun et al., 2020; Zhao et al., 2021). To our best knowledge, no study applying IRGPs to construct the prognostic signature in HNSCC. Here, we used TCGA datasets, GEO datasets, and immune-related genes from the ImmPort database to establish and validate the immune prognostic signature, which was constructed by 22 immune-related gene pairs consisting of 28 unique immune-related genes. The relationships of IRGPs with clinical features and underlying molecular function enrichments and immune cell proportions were further discovered in our study.

## Materials and Methods

### Acquisition of Relevant Public Data

The RNA-seq expression data and clinical data of 525 HNSCC patients in the training group were downloaded from the TCGA dataset^1^, which was used as the training cohort. Two independent datasets were used as the validation group were downloaded from the GEO dataset in this study, including GSE65858 (*N* = 270, Illumina HumanHT-12 V4.0 expression bead chip) (Wichmann et al., 2015) and GSE2379 (*N* = 34, Affymetrix Human Genome U95 Version 2 Array and Affymetrix Human Genome U95A Array) (Cromer et al., 2004). For each dataset, the probe ID was converted to the corresponding gene symbol according to its annotation file without making further standardization. If more than one probe matches the same gene, the average value is calculated as the expression value of the gene. The immune-related gene list was download from the ImmPort database^2^.

### Establishment and Validation of an Immune Gene-Related Prognostic Model

All the 2,498 immune-related genes from the ImmPort database were selected for the candidates’ immune-related genes to be intersected with the TCGA dataset and GEO dataset, respectively. The immune-related genes obtained from the above three cohorts are intersected to obtain a matrix that included 145 co-IRGs. During screen immune-related genes (IRG), the median absolute deviation must be greater than 0.5. Next, each gene will have a chance to be paired up with another gene in the matrix. The estimated score of the gene pair is up to the reduction from the gene counts. If the gene pair i is made up of gene_*i*_ and gene_*i*+1_. The score will be 1 if the expression count of gene_*i*_ is higher than gene_*i*+1_; otherwise, its score will be 0. More importantly, we should discard the gene if the percentage of the score was 1 or 0 was less than 20%. We used LASSO to get the maximum information but the parsimonious model (iteration = 1,000). The IRGPs’ signature was calculated as follows: Risk score = (Expr_*genepair*–1_ × Coef_*genepair*–1_) + (Expr_*genepair*–2_ × Coef_*genepair*–2_) + … + (Expr_*genepair*–*n*_ × Coef_*genepair*–*n*_). Expr means the expression value of gene pairs, and Coef means the regression coefficient derived. Then we used the TCGA survival data, risk score, and time-dependent ROC at 3 years in the TCGA dataset to screen the highest frequency IRGPs and calculate the cut-off value to determine the patient with high risk or low score. The capability of this model to predict overall survival (OS) was visualized and compared by Kaplan-Meier, univariate and multivariate Cox proportional hazard analysis. The external dataset GSE65858 and GSE22379 were used for the validations.

### Correlations Between IRGPs and Tumor-Infiltrating Immune Cells Proportions

The CIBERSORT algorithm, a method for characterizing cell composition from gene expression profiles of complex tissues (Newman et al., 2015), was used to explore the relationship between tumor immune cells and IRGPs. It should enable large-scale analysis for inferring immune cells, cellular biomarkers in tumor transcriptomes (Gentles et al., 2015). The proportions of 22 immune cell types were estimated in the TCGA cohort. A radar map is drawn for comparing these immune cell distributions between the patients with a high score and the ones with a low score according to the results of the CIBERSORT algorithm.

### Gene Enrichment Analysis

Gene ontology (GO) analysis was used to discover the enriched signaling pathway based on the genes in the IRGPs prognostic signature in HNSCC. Gene set enrichment analysis (GSEA) was used to understand the potential biological mechanisms these genes in IRGPs prognostic signature may involve.

### Statistical Analysis

All statistical analyses were performed using R (version 4.0.2^3^). The log-rank test was used in the Kaplan-Meier analysis. The ROC curve was performed by the “survivalROC” package. The “survival” package was used to perform survival analysis curves, univariate Cox survival analysis and multivariate Cox survival analysis. The radar map was drawn by the “fmsb” package. The GSEA curve and the bubble chart of GO enrichment analysis were performed by the “fgsea” and “ggplot2” packages. For all tests, the significant differences were considered at *P* < 0.05.

## Results

### Construction and Validation of IRGPs Signature in HNSCC

**FIGURE 1:**
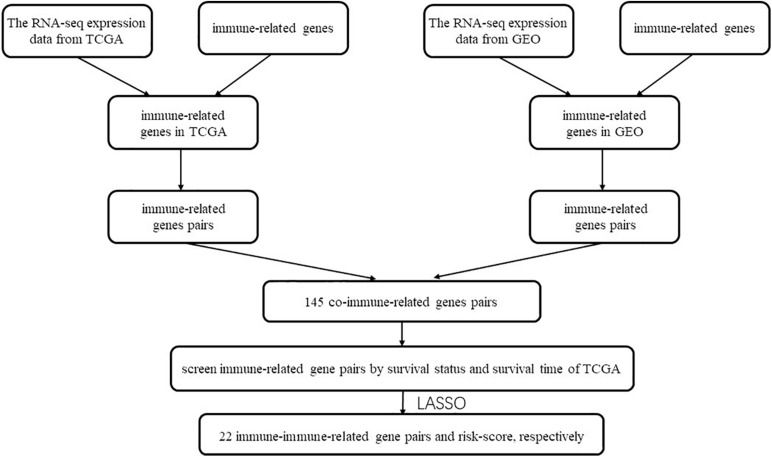
Flow chart of this study.

**FIGURE 2:**
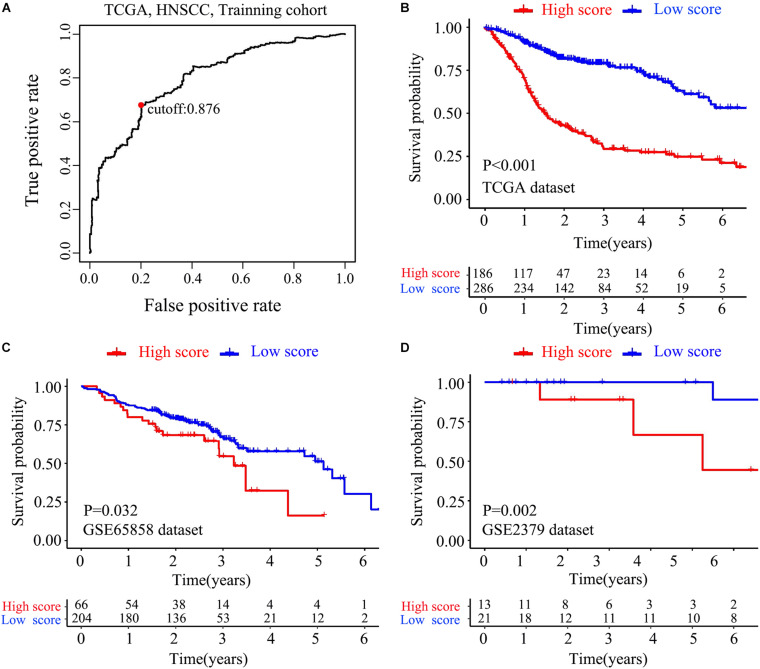
Construction and verification of IRGPs signature. **(A)** ROC curve analysis was used to get the best cut-off value of the score to divided patients into high score group or low score group. Kaplan-Meier survival curves assessed the effect between high score group and low score group in the TCGA dataset **(B)**, validated in GSE65858 **(C)** dataset and GSE22379 dataset **(D)** (*P* < 0.05).

The flow chart of this study is shown in Figure 1. The characterizations of the training and validation cohort are summarized in Table 1. As showing in Table 2, 22 highest frequency IRGPs were obtained from 2,498 immune-related genes in the ImmPort database after a couple of filtering mentioned above. The best cut-off value for the determination of high or low scores was calculated by ROC analysis in the TCGA dataset of HNSCC patients (sensitivity = 0.701, specificity = 0.812) (Figure 2A). The Kaplan Merrier analysis showed that patients with a high score of IRGPs had been predicted a poorer outcome as opposed to a low score of IRGPs (*P* < 0.001, Figure 2B), which was further validated in the GSE65858 cohort (*P* < 0.05, Figure 2C) and GSE2379 (*P* = 0.032, Figure 2D).

**TABLE 1:** Clinical parameters and IRGPs score in HNSCC cohorts.

Clinical parameters	TCGA data (*N* = 472)		GSE65858 (*N* = 270)		GSE2379 (*N* = 34)	
	Low score	High score	Low score	High score	Low score	High score
	(*N* = 286)	(*N* = 186)	(*N* = 204)	(*N* = 66)	(*N* = 21)	(*N* = 13)
**Age (years)**						
Mean (*SD*)	61.68	60.6	60	59	57.3	58.8
Median (min, max)	61 (24, 87)	61 (19, 90)	59 (35, 87)	57 (40, 82)	56 (41, 72)	58 (43, 70)
**Gender**						
Female	72 (25.2%)	53 (28.5%)	33 (16.2%)	14 (21.2%)	0 (0%)	2 (15.4%)
Male	214 (74.8%)	133 (71.5%)	171 (83.8%)	52 (78.8%)	21 (100%)	11 (84.6%)
**Tumor stage**						
T1–2	126 (44.1%)	57 (30.6%)	85 (41.7%)	30 (45.5%)	5 (23.8%)	5 (38.5%)
T3–4	157 (54.9%)	129 (69.4%)	119 (58.3%)	36 (54.5%)	16 (76.2%)	8 (61.5%)
Missing	3 (1%)	0	0	0	0	0
**Neck nodal metastasis**						
N-	133 (46.6%)	66 (36.5%)	67 (32.8%)	27 (40.9%)	3 (14.3%)	1 (7.7%)
N+	148 (51.7%)	119 (64.0%)	137 (67.2%)	39 (59.1%)	18 (85.7%)	12 (92.3%)
Missing	5 (1.7%)	1 (0.5%)	0	0	0	0
**Distant metastasis**						
M0	274 (95.9%)	181 (97.3%)	198 (97.1%)	65 (98.5%)	21 (100%)	13 (100%)
M1	2 (0.6%)	2 (1.1%)	6 (2.9%)	1 (1.5%)	0	0
Missing	10 (3.5%)	3 (1.6%)	0	0	0	0
**Grade stage**						
I–II	70 (24.5%)	27 (14.5%)	37 (18.1%)	18 (27.3%)	/	/
III–IV	216 (75.5%)	159 (85.5%)	167 (81.9%)	48 (72.7%)	/	/

**TABLE 2:** Information on the 22 immune-related gene pairs and the coefficient in HNSCC.

Gene pair1	Full name	Gene pair2	Full name	Coefficient
WNT5A	Wnt family member 5A	STC1	Stanniocalcin 1	–0.41604
HLA-DOB	Major histocompatibility complex, class II, DO beta	VEGFC	Vascular endothelial growth factor C	–0.22043
LTB4R	Leukotriene B4 receptor	HBEGF	Heparin binding EGF like growth factor	–0.17237
SPINK5	Serine peptidase inhibitor Kazal-type 5	NAMPT	Nicotinamide phosphoribosyltransferase	–0.13680
DEFB1	Defensin beta 1	HBEGF	Heparin binding EGF like growth factor	–0.07110
CD1A	CD1a molecule	DKK1	Dickkopf WNT signaling pathway inhibitor 1	–0.06338
SPINK5	Serine peptidase inhibitor Kazal type 5	IRF7	Interferon regulatory factor 7	–0.05752
SPINK5	Serine peptidase inhibitor Kazal type 5	STC2	Stanniocalcin 2	–0.00787
THBS1	Thrombospondin 1	CXCL9	C-X-C motif chemokine ligand 9	0.03613
FABP4	Fatty acid binding protein 4	VCAM1	Vascular cell adhesion molecule 1	0.07401
APOD	Apolipoprotein D	CD79A	CD79a molecule	0.07992
DKK1	Dickkopf WNT signaling pathway inhibitor 1	NR4A2	Nuclear receptor subfamily 4 group A member 2	0.09410
STC2	Stanniocalcin 2	NR1H3	Nuclear receptor subfamily 1 group H member 3	0.11060
HLA-DOB	Major histocompatibility complex, class II, DO beta	CD79A	CD79a molecule	0.12256
DKK1	Dickkopf WNT signaling pathway inhibitor 1	IL24	Interleukin 24	0.12949
APOD	Apolipoprotein D	VCAM1	Vascular cell adhesion molecule 1	0.12954
DKK1	Dickkopf WNT signaling pathway inhibitor 1	GHR	Growth hormone receptor	0.13036
CXCL1	C-X-C motif chemokine ligand 1	DEFB1	Defensin beta 1	0.16358
OASL	2′–5′-oligoadenylate synthetase like	CD79A	CD79a molecule	0.21155
CXCL1	C-X-C motif chemokine ligand 1	CCL5	C-C motif chemokine ligand 5	0.21247
FABP4	Fatty acid binding protein 4	PDGFRA	Platelet derived growth factor receptor alpha	0.21621
APOD	Apolipoprotein D	CD247	CD247 molecule	0.42530

**FIGURE 3:**
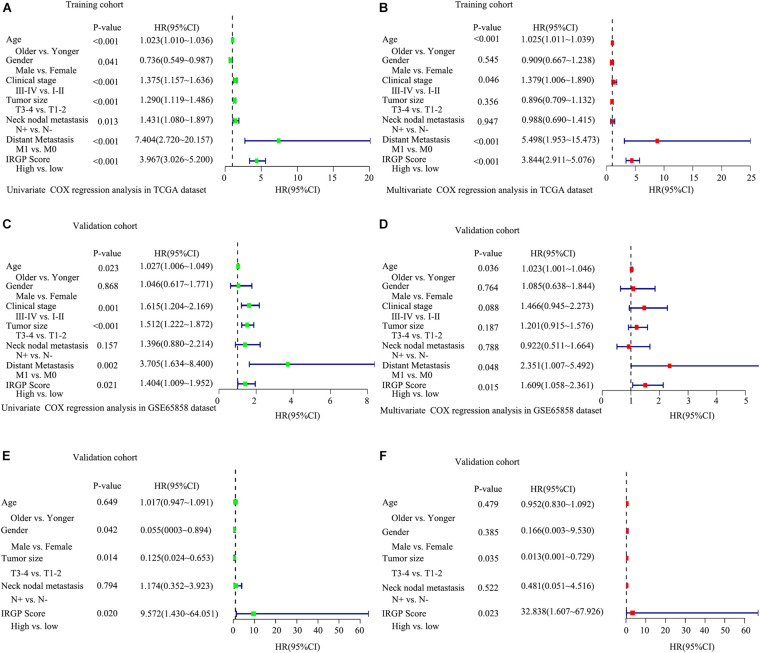
Explored and validation of independent clinical prognostic factors related to the signature of IRGPs in HNSCC. Univariate analysis and multivariate analysis were used to explored independent clinical prognostic factors in the TCGA dataset **(A,B)**, validated in GSE65858 dataset **(C,D)** and GSE2379 dataset **(E,F)**.

To test whether the effect of IRGPs on HNSCC prognosis is independent of clinical parameters like age, clinical stage, lymph node status, etc., univariable and multiple variable Cox proportional regression analysis were performed. The death hazard ratio of univariate regression analyses of a high score is independently and significantly higher over a low score in the training TCGA cohort (HR = 3.967, 95% CI 3.026–5.200, *P* < 0.001, Figure 3A), which was validated both in the GSE65858 cohort (HR = 1.404, 95% CI 1.009–1.952, *P* = 0.021, Figure 3C) and GSE2379 cohort (HR = 9.572, 95% CI 1.430–64.051, *P* = 0.02, Figure 3E). The same results in multivariate regression analysis. TCGA cohort (HR = 3.844, 95% CI 2.911–5.076, *P* < 0.001, Figure 3B), GSE65858 (HR = 1.609, 95% CI 1.058–2.361, *P* = 0.0115, Figure 3D) and GSE2379 (HR = 23.838, 95% CI 1.607–67.926, *P* = 0.023, Figure 3F).

### The Differential Tumor-Infiltrating Immune Cells Between HNSCC Patients With Different IRGPs

**FIGURE 4:**
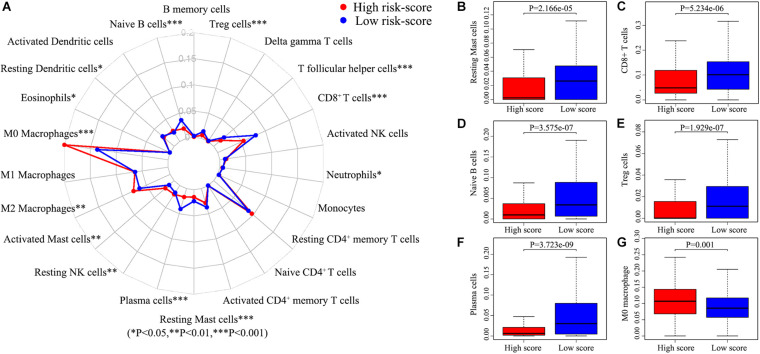
Immune cells correlation of IRGPs signature in HNSCC. **(A)** Using CIBERSORT calculated the abundance of 22 different immune cells in high score group and low score group of HNSCC. The abundance distribution of **(B)** Resting mast cells, **(C)** CD8^+^ T cells, **(D)** Naïve B cells, **(E)** Treg cells, **(F)** Plasma cells, and **(G)** M2 macrophages within the two groups (*P* < 0.05).

**FIGURE 5:**
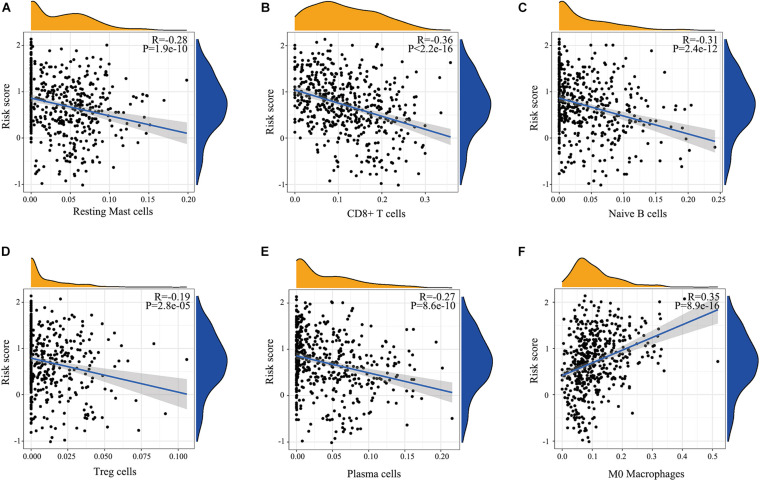
Relationship determined by Pearson correlation analysis between IRGPs and infiltration relative abundances of immune cells. **(A)** Resting mast cells, **(B)** CD8^+^ T cells, **(C)** Naïve B cells, **(D)** Treg cells, **(E)** T follicular helper cells, **(F)** Plasma cells.

There are many different proportions of immune cells found in HNSCC patients with high-score over low-score of IRGPs (Figure 4). As shown in the radar map (Figure 4A), we found that naïve B cells, Plasma cells, Resting mast cells, CD8^+^ T cells, and Treg cells were significantly enriched in patients with a low score of IRGPs than the high score of IRGPs (*P* < 0.001) (Figures 4B–F). However, M0 macrophages were significantly higher in the high score group than the low score group (Figure 4G). The comparisons about the other immune cells in the two groups of patients were shown in Supplementary Figure 1. Furthermore, we explored the potential associations between the IRGPs’ signature and 22 immune cells by Pearson correlation analysis. We found that the signature risk score was significantly and negatively correlated to resting mast cells (*R* = –0.28), CD8^+^ T cells (*R* = –0.36), naïve B cells (*R* = –0.31), Treg cells (*R* = –0.19), and plasma cells (*R* = –0.27) (Figures 5A–E, all *P* < 0.01). However, the signature risk score was positively correlated to M0 macrophages (Figure 5F, *R* = 0.35, *P* < 0.01).

#### Performance of IRGPs on Predicting HNSCC Prognosis

**FIGURE 6:**
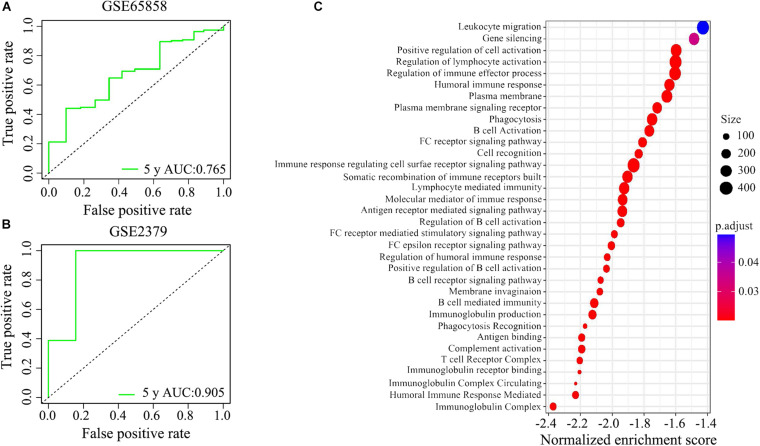
The potential signaling pathways of the signature of IRGPs were explored by GEO enrichment analysis. **(A,B)** The receiver operating characteristic (ROC) curve illustrated the predictive ability of the 22 IRGPs signature. The areas under the curves for 5 years’ survival were 0.765 and 0.905 in two validation cohorts, respectively. **(C)** The signature of IRGPs in the TCGA cohort of HNSCC was involved in the positive regulation of cell activation, regulation of lymphocyte activation, regulation of immune effector process, humoral immune response, plasma membrane, and phagocytosis.

We applied the ROC analysis to explore the predictive ability of the IRGPs’ signature on the 5-year overall survival in the two validated HNSCC cohorts. The value of under the curve (AUC) in GSE65858 and GSE2379 was 0.765 and 0.905, respectively (Figures 6A,B).

#### Enrichment Analysis Explored Potential Signaling Pathways

**FIGURE 7:**
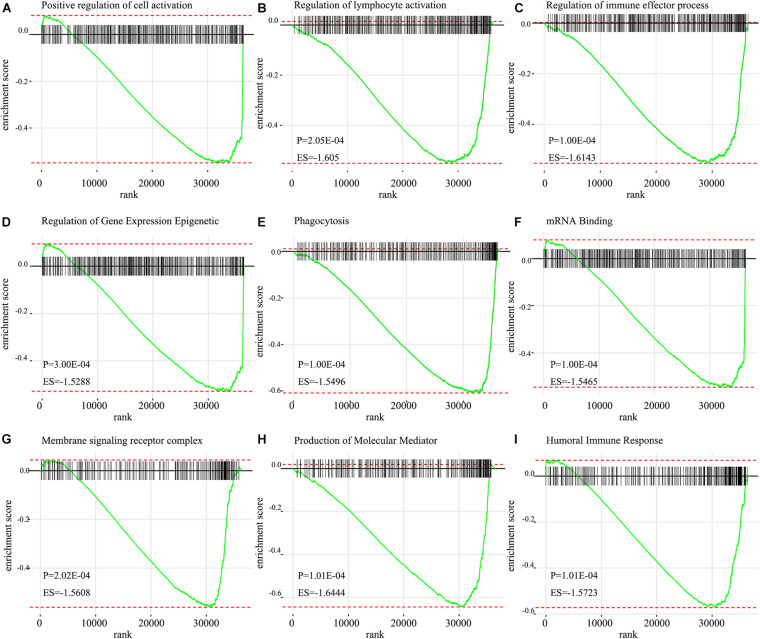
GSEA enrichment plots from gene set enrichment analysis. The results show that nine cancer hallmark gene set included **(A)** positive regulation of cell activation, **(B)** regulation of lymphocyte activation, **(C)** Regulation of immune effector process, **(D)** Regulation of gene expression epigenetic, **(E)** Phagocytosis, **(F)** mRNA binding, **(G)** Membrane signaling receptor complex, **(H)** Production of molecular mediator, **(I)** Humoral immune response are significant enrichment of low score of immune-related pathways in patients with HNSCC (*P* < 0.05).

Go analysis showed that the signature of IRGPs in the TCGA cohort of HNSCC was involved in the positive regulation of cell activation, regulation of lymphocyte activation, regulation of immune effector process, humoral immune response, plasma membrane and phagocytosis (Figure 6C). According to the GSEA analysis, we revealed positive regulation of cell activation, regulation of lymphocyte activation, regulation of immune effector process, regulation of gene expression epigenetic, phagocytosis, mRNA binding, membrane signaling receptor complex, production of molecular mediator, and humoral immune response (Figure 7). Based on their normalized enrichment score, a lot of enriched signaling pathways were identified. It mainly including regulation of cell activation, regulation of lymphocyte activation, regulation of immune effector process, phagocytosis, and humoral immune response, which may be the immune-related pathways of significant enrichments in HNSCC. It provides possible immunologic concepts to support the prediction of IRGPs for HNSCC prognosis.

## Discussion

HNSCC is the sixth most prevalent malignant tumor worldwide. The immune system is the main defense system of people against diseases, including cancers (Huse, 2017). Exploring the mechanisms between the immune-related genes and cancer prognosis may open a new perspective for predicting the outcomes of HNSCC patients. Currently, immunotherapy, receiving extensive attention all around the world, has been demonstrated as a groundbreaking remedy for cancer patients by blocking CTLA-4 or PD-1 pathways (Yang, 2015). Besides, researchers show that human peripheral blood T lymphocytes transduced by CD19-CAR could treat leukemia well (Brentjens et al., 2003). Therapeutic cancer vaccines and tumor neoantigens are other options for joining the armies of immunotherapy. Due to a lack of ideal vaccine design and the existence of an immunosuppressive tumor microenvironment, therapeutic vaccination remains suboptimal (Melief et al., 2015).

Continued efforts to understanding the role of the immune microenvironment will provide better methods for the treatment of HNSCC. So, we applied 22 immunes relate gene pairs to construct a prognostic signature and further explore its potential role in HNSCC prognosis. A previous study has reported that immune-related genes provide a model for predicting outcomes in HNSCC (She et al., 2020). However, a limitation is the lack of robust normalization to reduce batch effects if different platforms or sources are involved. Therefore, the first step needs to standardize all genes to reduce the errors caused by different samples and platforms. To overcome this limitation, we adopted a method named IRGPs (validated in a variety of cancers; Peng et al., 2018) to assess the score of the sample by using the comparable relative score of a pair of gene expressions in the same sample, which could remove the biases from the different platforms and samples.

In this study, 515 HNSCC patients from TCGA datasets act as the training group, the data of 270 patients from GSE65858 and 34 patients from GSE2379 act as the validation groups. Lasso penalized Cox regression was used to construct a 22-IRGPs signature and ROC curve analysis was used to calculate the best cut-off value. The HNSCC patients with a high or low score of IRGPs have a significant difference in survival analysis. Univariate and multivariate Cox regression analyses showed the high score was an independent prognostic factor. Besides, we used two external cohorts to confirm the conclusions, which remain the same as in the training group. Consequently, our signature has high accuracy and could be a potential prognostic factor in HNSCC. We found that naïve B cells, Plasma cells, Resting mast cells, CD8^+^ T cell, and Treg cells were significantly enriched in patients with a low score of IRGPs by immunocyte infiltration analysis, which may play an inhibitory role in the development of HNSCC. It has report showed that the proportions of naïve B cells, resting mast cells, and CD8^+^ T cells in non-cancer tissues were significantly higher than HNSC tissues (Krishna et al., 2018). It is further confirmed the accuracy of the results of our study. Furthermore, some research also found that plasma cells were associated with distant cancer metastasis, clinical stage. Naïve B cells and Tregs were significantly associated with pathological grade (Liang et al., 2020). These differential proportions of tumor-infiltrating cells may be reasons for the different overall survival observed and validated between a high and low score of IRPGs and associated with the IRGP interactions in HNSCC patients.

The 22 IRGPs in our model consisted of 28 immune-related genes, including Wnt5a, CCL1, CCL5, CCL9, HLA-DOB, IFN, and CD247, which mainly involved some important pathways and cellular processes such as proinflammatory, antimicrobial responses, antigen identification, and immune cell signaling. Wnt signaling regulates a series of signaling pathways, including cell proliferation, differentiation, adhesion, and motility (Wend et al., 2010). Wnt5a act as a non-canonical Wnt ligand that plays a critical role during growth and development (Oishi et al., 2003). The abnormal activation and inhibition of Wnt5a lead to the genesis in tumors. Increased Wnt5a level promotes the ability of invasion in metastasis melanoma (Weeraratna et al., 2002). In contrast, Wnt5a acts as a potential inhibitor. The increased Wnt5a level inhibits cell growth and migration in thyroid carcinoma (Kremenevskaja et al., 2005). Furthermore, several reports have already suggested that Wnt5a plays an important role in cancer-associated inflammation. Wnt5a induced gastric epithelial cells to produce IL-1, leading macrophages gathered in gastric mucosa inducing gastric inflammation (Asem et al., 2016). Most of the research reported that chemokines and their receptors play an important role in mediated cancer progress and metastasis. For instance, CCL1 derived from tumor-associated macrophages promotes breast cancer metastasis through activating NF-κB/SOX4 signaling (Wang et al., 2018). CXCL9 predominantly mediates lymphocytic infiltration to the focal sites and suppresses tumor growth (Tokunaga et al., 2018). The knockout of NLRX1 could significantly decrease IFN-I dependent T cell infiltration and inhibit tumor progression (Luo et al., 2020). HLA-DOB consists of a class II molecule and a beta chain and expresses on the antigen-presenting cells and B lymphocytes (Pruitt et al., 2009). The above descriptions indicated that immune response contributes to the development and progression of carcinomas in the interaction of the immune gene. It is consistent with our results that IRGPs were significant associated with some kinds of immune cells, including CD8^+^ T cells, Treg cells, and Resting mast cells (Li et al., 2021). The immune-related genes inside our IRGPs might play an important role in the tumor microenvironment of HNSCC. The GSEA and GO enrichment analysis implied that regulation of positive regulation of cell activation, regulation of lymphocyte activation, regulation of immune effector process, regulation of gene expression epigenetic, phagocytosis, mRNA binding, membrane signaling receptor complex, production of molecular mediator and humoral immune response might be the significant enrichment of related pathways in HNSCC, which give us more confidence in the capability of IRGPs to predict the HNSCC prognosis as we all know that the expression of gene reduced can promote most of diseases development. For example, mice with the knockout of EIF4E-BP1, a direct target of mTOR, formed more and larger lesions than its wild type mice (Wang et al., 2019). The humoral immune response is an important part of the immune system. Since tumors originate from containing self-antigens of autologous cells, the abnormal exposure of these antigens can activate the autoimmune response to promote the development of cancer (Zaenker et al., 2016). Therefore, we suggested that the 22 IRGPs signature was constructed in our research act as an important role in the development and prognosis of HNSCC. The reason that we chose two datasets GSE65858 and GSE22379 as validation cohorts is that these two databases of HNSCC contain the most complete clinical data. Although the second database contains a relatively small number of patients, it contains comprehensive patients’ information such as survival time, survival status, and TMN staging that we want. The clinical data of age in the multivariate COX regression analysis among three groups are not in the same range, *P* < 0.001, =0.036, =0.479, respectively, in Figures 3B,D,F. Therefore, we don’t think age can be used as a prognostic factor for HNSCC.

However, we are obliged to admit the limitations of our research. Firstly, although we used the validation group to prove the training group, the number of samples is not large enough. Secondly, it can be more persuasive if we further validate our findings through RT-QPC or IHC.

In conclusion, we established a new IRGPs signature for predicting the prognosis in HNSCC, which may provide a possible tool to help clinical physicians to give HNSCC patients consult about the prognosis.

## Conclusion

We developed a new IRGPs signature prognostic model in HNSCC. It probably confers a new insight into the prognosis prediction of HNSCC patients, which may provide some clues for future personalized medicine decisions.

## Data Availability Statement

The raw data of this study are derived from the TCGA database (https://portal.gdc.cancer.gov/) and GEO data portal (https://www.ncbi.nlm.nih.gov/geo/; Accession Nos.: GSE65858 and GSE2379), which are publicly available databases. The R codes will be available upon request.

## Author Contributions

MH: conception, revision for important intellectual content, and supervision. JH: interpretation or analysis of data. XF: preparation of the manuscript. All authors contributed to the article and approved the submitted version.

## Conflict of Interest

The authors declare that the research was conducted in the absence of any commercial or financial relationships that could be construed as a potential conflict of interest.

## Footnotes

1 https://portal.gdc.cancer.gov/

2 https://immport.niaid.nih.gov/home

3 https://www.r-project.org/

## Supplementary Material

Supplementary Figure 1 Any other immune cells enriched of IGRPs signature in HNSCC.
