# Supplementary figures and images for: Discovery and Verification of an Immune-Related Gene Pairs Signature for Predicting Prognosis in Head and Neck Squamous Cell Carcinoma
Source: Front Genet. 2021 May 24;12:654657. doi: 10.3389/fgene.2021.654657 (PMC8181401; doi:10.3389/fgene.2021.654657)

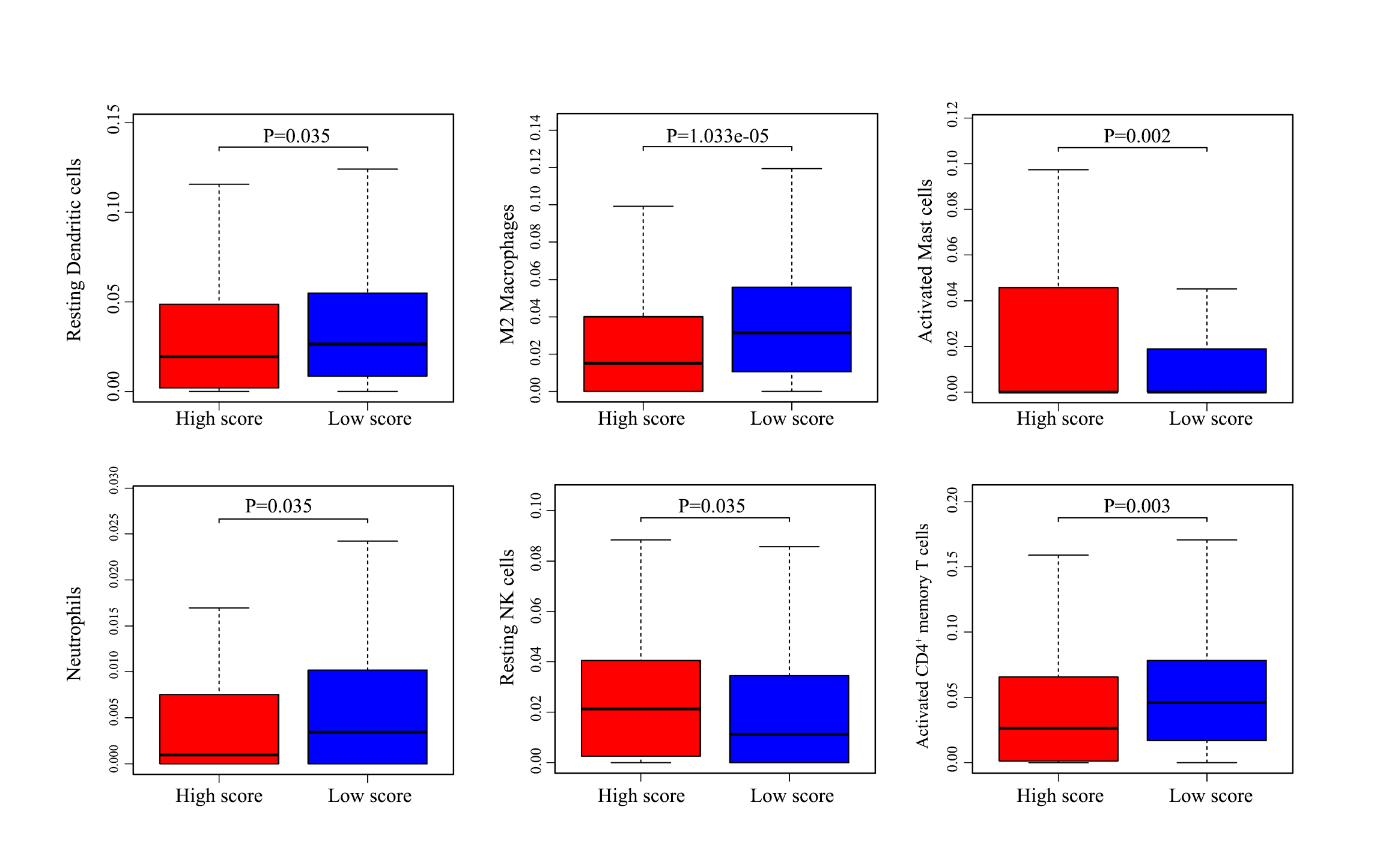

Supplement: Supplementary Figure 1 — Any other immune cells enriched of IGRPs signature in HNSCC. [file Image_1.JPEG]
